# Genome Editing in Rice: Recent Advances, Challenges, and Future Implications

**DOI:** 10.3389/fpls.2018.01361

**Published:** 2018-09-19

**Authors:** Rukmini Mishra, Raj Kumar Joshi, Kaijun Zhao

**Affiliations:** ^1^National Key Facility for Crop Gene Resources and Genetic Improvement, Institute of Crop Science, Chinese Academy of Agricultural Sciences, Beijing, China; ^2^Department of Biotechnology, Rama Devi Women’s University, Bhubaneswar, India

**Keywords:** base editors, crop improvement, CRISPR/Cas9, CRISPR/Cpf1, genome editing, rice, targeted mutagenesis

## Abstract

Rice (*Oryza sativa* L.) is the major food source for more than three billion people of the world. In the last few decades, the classical, mutational, and molecular breeding approaches have brought about tremendous increase in rice productivity with the development of novel rice varieties. However, stagnation in rice yield has been reported in recent decade owing to several factors including the emergence of pests and phyto pathogens, climate change, and other environmental issues posing great threat to global food security. There is an urgent need to produce more rice and associated cereals to satisfy the mammoth task of feeding a still growing population expected to reach 9.7 billion by 2050. Advances in genomics and emergence of multiple genome-editing technologies through use of engineered site-specific nucleases (SSNs) have revolutionized the field of plant science and agriculture. Among them, the CRISPR/Cas9 system is the most advanced and widely accepted because of its simplicity, robustness, and high efficiency. The availability of huge genomic resources together with a small genome size makes rice more suitable and feasible for genetic manipulation. As such, rice has been increasingly used to test the efficiency of different types of genome editing technologies to study the functions of various genes and demonstrate their potential in genetic improvement. Recently developed approaches including CRISPR/Cpf1 system and base editors have evolved as more efficient and accurate genome editing tools which might accelerate the pace of crop improvement. In the present review, we focus on the genome editing strategies for rice improvement, thereby highlighting the applications and advancements of CRISPR/Cas9 system. This review also sheds light on the role of CRISPR/Cpf1 and base editors in the field of genome editing highlighting major challenges and future implications of these tools in rice improvement.

## Introduction

The rice (*Oryza sativa* L.) grain makes up 20% of the world’s dietary energy supply and more than three billion people across the globe uptake rice daily (Birla et al., 2017). Due to its wider adaptability under different environmental conditions, rice has been regarded as a IS strategic crop for food security worldwide by the Food and Agriculture Organization (FAO) (Montano et al., 2014). Global rice consumption is projected to increase from 450 million tons in 2011 to about 490 million tons in 2020 and to around 650 million tons by 2050 (Rejesus et al., 2012). It has been estimated that 40% more rice is needed to be produced by 2050 to meet the food demands of the ever increasing population (Milovanovic and Smutka, 2017). In the last few decades, conventional, mutational, and molecular breeding approaches have contributed enormously toward enhanced rice productivity. However, the rice yield is gradually declining in recent decades. Currently, agriculture is facing multiple challenges such as rapid population growth, global climate change, emergence of pests and pathogens, and other environmental hazards. Thus, there is an urgent need to have more advanced and improved technologies that can develop new rice varieties with higher yield potential, enhanced abiotic stress tolerance, and improved resistance to major pests and pathogens.

The recent emergence of genome editing technologies have superseded the limitations of traditional breeding methods starting a new era of crop improvement. Genome editing involves the usage of engineered site-specific nucleases (SSNs) to modify specific genes at desired locations in the genome. The SSNs such as zinc finger nucleases (ZFNs), transcriptional activator-like effector nucleases (TALENs) and clustered regularly interspaced short palindromic repeats (CRISPR)-associated endonuclease Cas9 (CRISPR/Cas9) make a double-stranded break (DSB) in the target DNA which is subsequently repaired by cell’s own natural repair mechanism of homologous recombination (HR) or non-homologous end joining (NHEJ) (Miglani, 2017). The NHEJ repair is the error prone pathway which creates random insertions and deletions (indels) and results in frame shift mutations and targeted gene knockouts (Feng et al., 2013; Bortesi and Fischer, 2015), whereas the HR pathway is much more precise in the exchange of homologous sequence leading to gene knock in or gene replacement (Voytas and Gao, 2014; Baltes et al., 2014).

Clustered regularly interspaced short palindromic repeats-associated endonuclease Cas9 is the most advanced genome editing tool in plant biology (Belhaj et al., 2015; Weeks et al., 2016). It consists of a short RNA molecule called guide RNA which is associated with a DNA endonuclease called Cas9. CRISPR-associated protein 9 (Cas9) is a DNA endonuclease responsible for cutting the invading phage DNA into pieces, which then gets integrated into the CRISPR array as a spacer. The guide RNA is a two-component system consisting of the crRNA (CRISPR-derived RNA) and tracrRNA (trans-activating RNA). In nature, the crRNA targets the double stranded DNA to be cut, and has a short region of homology allowing it to bind the tracrRNA. The tracrRNA provides a stem loop structure which associates with Cas9 protein. In the CRISPR/Cas9-based genome editing system, the crRNA and tracrRNA were engineered into a single guide RNA chimera (sgRNA) that can also direct sequence-specific Cas9 dsDNA cleavage (Jinek et al., 2012). The protein/RNA complex (Cas9-sgRNA) moves along the DNA strand and makes a double stranded break (DSB) where the sgRNA matches the target DNA sequence (Jinek et al., 2014). The CRISPR/Cas9 and its modified versions have wide applications in animals, plants, yeast, and human as well as in non-human cell lines (Doudna and Charpentier, 2014; Khatodia et al., 2016; Miglani, 2017). CRISPR/Cas9 system has been successfully used in major crops and model plants due to its simplicity, adaptability, and high precision (Ma X. et al., 2015; Xu et al., 2016).

The recent identification of another class 2 CRISPR effector, *Cpf1*, has broadened the horizon for genome editing and have strengthen the agricultural research (Zetsche et al., 2015). Most recently, the base editing technology has emerged as a new approach which overcomes some of the limitations of NHEJ and HR methods and converts one target base into another without the requirement of a DSB or donor template (Komor et al., 2016).

Rice is an excellent model system for functional genomics studies due to its small genome size, availability of genetic resources, high transformation efficiency, and greater genomic synteny with other cereals. Therefore, rice has been increasingly used to test the efficiency of different types of genome editing technologies (Li et al., 2012; Feng et al., 2013), to study the functions of various genes and demonstrate their potential in rice improvement (Xu et al., 2014, 2016; Wang et al., 2016). Although several reviews on genome editing and its role in plants have been published in the recent times (Bortesi and Fischer, 2015; Khatodia et al., 2016; Arora and Narula, 2017; Mohanta et al., 2017; Zhang et al., 2017; Mishra and Zhao, 2018), an elaborative review particularly on genome editing of rice is the need of the hour keeping in view the rapid and large accumulation of case studies on genome editing of this agriculturally important crop. The application of genome editing tools have broaden rice research, bringing in new opportunities to develop novel varieties with improved productivity and quality. In the present review, we focus on the different genome editing strategies and their applications in rice improvement using specific case studies. We have also highlighted the emergence of CRISPR/Cpf1 system and base editing as a suitable alternative to traditional CRISPR/Cas9 system for rice improvement. Furthermore, the review also focusses on the major challenges and future implications of genome editing in rice improvement.

## Concept-Proof Demonstration of Genome-Editing in Rice

### TALENs and CRISPR/Cas9

In last 5 years, researchers have published numerous articles demonstrating successful targeted mutagenesis in a wide range of crops using TALENs, CRISPR/Cas9, and CRISPR/Cpf1 systems (Jiang et al., 2013; Miao et al., 2013; Shan et al., 2013; Endo M. et al., 2016; Yin et al., 2017). Targeted mutagenesis in rice has been reported as early as 2012 when the rice bacterial blight susceptibility gene *Os11N3* (also known as *OsSWEET14*) was targeted for TALEN-based disruption producing disease resistant rice lines (Li et al., 2012). Subsequently, many such studies involving the use of TALENs for targeting multiple susceptible genes has been carried out to confer broad spectrum resistance against blight disease in rice (Hutin et al., 2015; Blanvillain-Baufum et al., 2017; Cai et al., 2017). Besides bacterial leaf blight (BLB), TALEN technology was used to disrupt the *Oryza sativa* betaine aldehyde dehydrogenase 2 (*OsBADH2*) gene for enhanced fragrance in rice (Shan et al., 2015). In a study, it was demonstrated that Lig4 plays an important role in the cNHEJ pathway in rice plants and lack of DNA Ligase4 or lig4 knockout rice lines can enhance the frequency of TALEN-mediated targeted mutagenesis in rice (Nishizawa-Yokoi et al., 2016).

In 2013, five major research articles were published reporting successful targeted mutagenesis by CRISPR/Cas9 system in rice (Feng et al., 2013; Jiang et al., 2013; Miao et al., 2013; Shan et al., 2013; Xie and Yang, 2013). A single customized sgRNA was used to introduce target mutations in three rice genes, *OsBADH2*, *Os02g23823*, and *OsMPK2* resulting in higher mutation frequency of CRISPR/Cas9 system as compared to TALENs (Shan et al., 2013). For BLB resistance, CRISPR/Cas9 construct designed for *OsSWEET14* and *OsSWEET11* resulted in the deletion of nine and seven nucleotides from the promoter region of the *OsSWEET14* and *OsSWEET11* genes (Jiang et al., 2013). In the same year, a CRISPR/Cas9 construct was used for simultaneous targeting of three rice genes, *rice outermost cell-specific gene 5* (*ROC5*), *stromal processing peptidase* (*SPP*) and *young seedling albino* (*YSA*), resulting in homozygous or bi-allelic mutants with the mutation frequency as high as 84% in the T_0_ and T_1_ rice lines (Feng et al., 2013). Additionally, four sugar efflux transporter genes (*OsSWEET11*, *OsSWEET12*, *OsSWEET13*, and *OsSWEET14*) were targeted by CRISPR/Cas9 vectors resulting in large chromosomal deletions between two nuclease-targeted loci (Zhou et al., 2014). As such, the constructs were suggested to be used for knockout screening of the entire rice genome with sgRNA libraries that can result in mutant rice populations with greater heritable variability and precision. These studies clearly indicate that CRISPR/Cas9 system can be used as an effective tool for chromosomal engineering, production of insertion, deletion, substitution, and translocation lines exhibiting greater efficiency for the development of new cultivars with improved novel traits.

Multiparalogous gene knockout was successfully achieved in rice by utilizing off target mutations *via* the CRISPR/Cas9 system (Endo M. et al., 2016). Targeted mutation of three rice genes, namely, *phytoene desaturase* (*OsPDS*), *Os02g23823*, and *OsMPK2* revealed a high co-mutation rate with the mutation frequency ranging between 66.4 and 81%. In another study, a high-efficiency multiplex genome editing was attempted in rice by generating multiple sgRNA cassettes (Ma X. et al., 2015). As many as 46 target sites were edited in the rice genome with an average mutation frequency of 85.4%. The study also demonstrated simultaneous editing of three sites within the *OsWaxy* gene, resulting up to 14% reduction in amylose content. Multiplex genome editing was also reported by using an endogenous tRNA processing system in rice, where each sgRNA was flanked by tRNA and processed into single sgRNAs resulting in large deletions of genomic sequences in T_0_ generation (Xie et al., 2015). Similarly, Liang et al. (2016) developed a new strategy for CRISPR/Cas9-sgRNA multiplex editing system in rice wherein 21 sgRNAs were designed and the corresponding Cas9/sgRNAs expression vectors were constructed. Transformed rice plants were significantly edited and 82% of the desired target sites represented deletion, insertion, substitution, and inversion, thereby displaying high editing efficiency. All these reports clearly indicate that the CRISPR/Cas9 system is highly efficient to generate multiple gene mutations using conventional strategy that could be subsequently used for the acceleration of rice breeding in future.

### CRISPR/Cpf1

**FIGURE 1:**
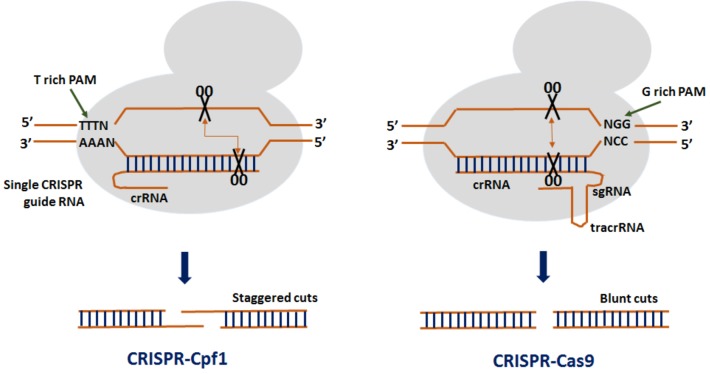
Comparison between CRISPR from *Prevotella* and *Francisella* 1 (Cpf1) and CRISPR-Crisper associated protein 9 (Cas9). In a CRISPR–Cpf1 system, a T-rich protospacer adjacent motif (PAM) creates a double stranded break (DSBs) at the distal region of the recognition site producing cohesive ends. In a CRISPR–Cas9 system, a G-rich PAM creates DSBs toward the proximal end of the recognition site resulting in blunt ends.

Clustered regularly interspaced short palindromic repeats from *Prevotella* and *Francisella* 1 (CRISPR/Cpf1) is the advanced genome editing system which is being used for plant gene editing since 2016 (Endo A. et al., 2016). CRISPR/Cpf1 system has some important advantages over CRISPR/Cas9 which makes it more advance and efficient genome editing tool (**Figure 1**). Unlike CRISPR/Cas9 which requires a G-rich (5′-NGG-3′) PAM sequence at the 3′ end of the target, CRISPR/Cpf1 recognizes a T-rich (5′-TTTN-3 or 5′-TTN-3′) PAM at the 5′ end of the target sequence (Zetsche et al., 2015), thereby resulting in high cleavage efficiency. In CRISPR/Cpf1 system, Cpf1–crRNA complex itself can efficiently cleave the target DNA without the need of tracrRNA. As such, a crRNA of 40–45 nt long containing the repeat and the spacer is well enough to facilitate gene editing as compared to ˜100 nt sgRNA used in CRISPR/Cas9 system (Zetsche et al., 2015). The Cpf1 also exhibit dual enzymatic activity acting as RNAase to process the pre-crRNA to crRNA while its nuclease activity cleaves the double stranded DNA. Therefore, multiple crRNAs could be generated in the CRISPR/Cpf1 system driven by a single promoter making it simpler than CRISPR/Cas9. Also, Cpf1 contains a RuvC domain and a nuclease domain which cleaves the target and the secondary strands of the DNA at 23 and 17 bp, respectively, in the downstream of the PAM sequence generating cohesive end with 5-bp overhangs (Zetsche et al., 2015). As such, the generation of larger mutations increases the efficiency of HDR-mediated donor gene insertion at a desired genome location. The off-target activity with Cpf1 cleavage is also comparatively lower than Cas9. These distinct features make it more advanced and a potential genome editing tool (Zaidi et al., 2017). Currently, three CRISPR/Cpf1 systems (FnCpf1, LbCpf1, and AsCpf1) are available that have been used for genome editing in rice (Hu et al., 2017; Tang et al., 2017; Xu et al., 2017; Wang et al., 2017).

Clustered regularly interspaced short palindromic repeats from *Prevotella* and *Francisella* 1 system generated stable and heritable mutations in two endogenous rice genes *OsPDS* and *OsBEL* (Xu et al., 2017). The mutation efficiency ranged from 13.6 to 43.1% suggesting efficient application of this system in developing targeted mutants in rice. In a separate study, dual RNA polymerase II promoter expression system was used in CRISPR/Cpf1 gene editing approach to determine the activity of Cpf1 from *Acidaminococcus* sp. BV3L6 (AsCpf1) and *Lachnospiraceae* bacterium ND2006 (LbCpf1) and study their role in targeted gene repression *in vivo* (Tang et al., 2017). Results revealed that LbCpf1 coupled with Pol II-promoter and a double ribozyme system could be effectively used in the maturation and expression of crRNAs leading to effective mutagenesis in rice. CRISPR/Cpf1 system has been recently standardized for multiplex gene editing in rice (Wang et al., 2017). Engineered CRISPR/Cpf1 system using *Francisella novicida* Cpf1 (FnCpf1) and *Lachnospiraceae* bacterium ND2006 Cpf1 (LbCpf1) with a simple direct repeat (DR) guide array was able to generate independent mature DR induced mutations in transgenic plants. FnCpf1 was used to edit four members related to receptor such as kinases (*OsRLKs*) with a mutation frequency ranging from 43.8 to 75%. Similarly, LbCpf1 could edit four *OsBEL* genes of the CYP81A family at a frequency of 40–60%. The study indicates that the flexibility of the DR sequence of Cpf1 can help in optimizing the DR guide array which can further simplify the multiplex gene editing in plants.

In a subsequent study, CRISPR-based editing using FnCpf1 and LbCpf1 was independently adopted to check their efficiency in inducing HDR-mediated donor gene insertion (Begemann et al., 2017). The study demonstrated that both FnCpf1 and LbCpf1 could generate HDR-mediated targeted insertion and subsequent knockout of the *Chlorophyllide-a oxygenase* (*CAO1*) gene in rice. A preliminary application of this strategy was visible when CRISPR/Cpf1 technology was used to knock out an early developmental gene *EPFL9* (epidermal patterning factor like-9) in rice leading to eightfold reduction in stomatal density without any off-target activity. Most recently, two modified LbCpf1 variants were generated to investigate their genome editing efficiency and expand the range of CRISPR/Cpf1 system in rice (Li et al., 2018). The study reported that the LbCpf1 (RR) variant enables multiplex editing of target genes containing TYCV PAMs in rice. This is highly significant as it has ability to broaden the range of genome editing by targeting and editing the genome sequences containing non-canonical PAMs. Therefore, CRISPR/LbCpf1 (RR) variant of editing system could be universally used in basic plant research and crop breeding in the future.

### Base Editors

Although CRISPR/Cas9 and CRISPR/Cpf1-based genome editing in plants *via* homology directed repair (HDR) can be a feasible approach for gene replacement, the frequency and efficiency of template DNA delivery and targeted insertion or gene replacement is quite low. As an alternative, a CRISPR/Cas9-based base editor technology is the latest and most advanced approach which enables direct and irreversible conversion of one target base into another without the requirement of a DSB or donor template (Komor et al., 2016; Nishida et al., 2016). The base editor is a fusion of catalytically inactive Cas9 domain and a cytosine deaminase domain that converts G-C base pairs to A-T base pairs (Li J. et al., 2017; Lu and Zhu, 2017; May, 2017). Base editing approach has been efficiently optimized and demonstrated in cereal crops including rice, wheat and maize (Zhong et al., 2017). A precise base editing construct designated as nCas9-PBE, composed of rat Cytidine deaminase APOBEC1 and a Cas9 variant Cas9-D10A nickase (nCas9) was used for editing *OsCDC48* gene which regulates senescence and cell death (Huang et al., 2016); the base edited plants revealed a mutation efficiency of 43.48%. The results indicated that the nCas9-PBE can be efficiently used for site-specific C to T base editing in rice. Later on, targeted point mutation in rice was reported by the use of nCas9 fused with a *cytidine deaminase* enzyme (Li J. et al., 2017). A single site from the *phytoene desaturase* (*OsPDS*) gene and two sites from the *OsSBEIIb* gene of rice were selected for the purpose. The results showed distinct introduction of precise point mutations at all three target sites with a mutagenic efficiency more than 40%.

Another base editing approach involved the use of rat cytidine deaminase enzyme (APOBEC1) to check its feasibility for inducing point mutations in two agriculturally important genes *NRT1.1B* and *SLR1* in rice (Lu and Zhu, 2017). *NRT1.1B* gene encodes a nitrogen transporter and SLR1 gene encodes a DELLA protein. As per previous reports, a C/T replacement (Thr327Met) in *NRT1.1B* gene could increase nitrogen use efficiency in rice (Hu et al., 2015) and an amino acid substitution in or near its TVHYNP motif results in reduced plant height (Asano et al., 2009; Hu et al., 2015). A CRISPR/Cas9-APOBEC1 base editing system was used to target one site each from *NRT1.1B* and *SLR1* gene. Results showed 1.4–11.5% C/T substitution while 1.6–3.9% of the edited plants accounted for C/G replacement. More recently, targeted base editing has been reported in rice by introducing multiple herbicide-resistance point mutations through multiplex base editing (Shimatani et al., 2017). The researchers used a target-activation induced cytidine deaminase (Target-AID) with a construct comprising nuclease-deficient Cas9 (*dCas9*) or nickase CRISPR/Cas9 (*nCas9*) fused to *Petromyzonmarinus* cytidine deaminase (*PmCDA1*)1 and sgRNAs. Point mutation in acetolactate synthase (ALS) confers herbicide resistance in plants (Yu and Powles, 2014). In rice, the C287T mutation of *ALS* homolog gene results in an A96V amino acid substitution that confers resistance to the herbicide imazamox (IMZ).

A CRISPR/Cas9 toolkit comprising of rBE3 and rBE4 (rice base editors) was reported for efficient targeted base editing to induce genetic variations in rice (Ren et al., 2017). In this study, they have first codon-optimized rat *APOBEC1* gene and *UGI* gene of *Bacillus subtilis* bacteriophage PBS1, and then attached them to *Cas9n* gene at both ends with XTEN linker sequence and nuclear localization signal (NLS) sequence, respectively. The *APOBEC1-XTEN-Cas9n-UGI-NLS* chimeric gene, named rBE3, was expressed under the control of the CaMV35S promoter in rice leaf sheath protoplasts together with *OsCERK1*-targeting sgRNA transcribed from a rice U6 promoter. Following this study, the researchers further tried to optimize the rBE system with human AID (hAID) for introducing point mutations at target regions (Ren et al., 2018). The *hAID*^∗^Δ-*XTEN-Cas9n-UGI-NLS* gene (termed rBE5) was first tested in rice leaf sheath protoplasts, with the sgRNAs targeting a GCAC-containing *ApaL1* restriction site in *OsRLCK185* and a TCC-containing *Bam HI* restriction site in *OsCERK1* gene, respectively. Sequencing results revealed mutations with high frequency for C/T conversions suggesting *hAID*^∗^Δ-*XTEN-Cas9n-UGI-NLS* gene (termed rBE5) functions well on G/C, A/C, and T/C substitution in rice cells. They have further expanded the toolkit with pUbi: rBE9 vectors which harbor an engineered hyperactive hAID mutant version. Furthermore, AID^∗^Δ showed highly enhanced base editing efficiency in generating both gain-of-function and loss-of-function mutants in rice. Thus, the base editor toolkit has the potential to be widely applied in both biological studies and molecular breeding of rice in the future.

**FIGURE 2:**
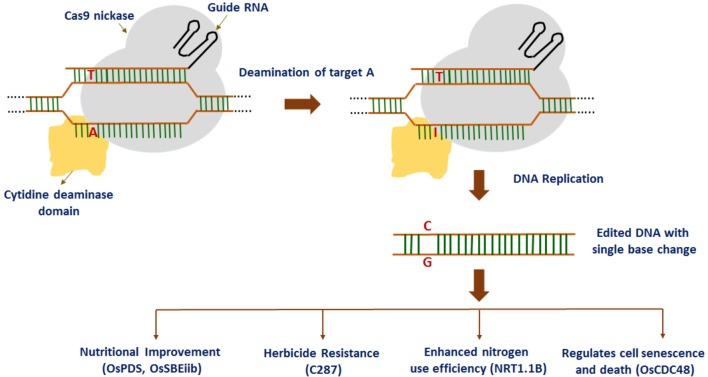
Recent advances in base editing technology in rice: point mutations are introduced into rice plants for nutritional improvement, herbicide resistance, enhanced nitrogen use efficiency, and regulating cell senescence and death.

Most recently, a fluorescence-tracking adenine base editor has been developed using the Cas9n-guided TadA: TadA7.10 heterodimer, efficiently introducing an A to G conversion in rice (Yan et al., 2018). At first, the wild-type *E. coli TadA* gene (the non-catalytic monomer) and *TadA^∗^7.10* version (the catalytic monomer) were codon-optimized and fused to the N-terminus of Cas9 (D10A) nickase (Cas9n) and catalytically deadCas9 protein (dCas9) with two XTEN2 linkers, thereby resulting in rBE14 and rBE15 base editors. Similarly, rice base editors rBE17 and rBE18 were created by importing A142N and P152R mutations into TadA^∗^7.10 to generate TadA^∗^7.8. Later on, rBE14, rBE15, rBE17, and rBE18 together with a *sgRNA* were introduced to target the pathogen-responsive phosphorylation site in the endogenous *OsMPK6* gene into rice cells to investigate the feasibility and efficiency of the adenine base editors. The study indicates that rBE14, together with the other rBE vectors, have the potential to facilitate generation of DNA variations in rice for both functional genomics and crop improvement. Overall, these findings suggest that base editing technologies can confer precise gain-of-function or loss-of-function which is crucial to accelerate the improvement of rice and other crops. In addition, precise A/T to G/C base editing has been reported in rice by using ABE7-10 base editors (Hua et al., 2018). IPA1 (*OsSPL14*), an important gene for plant architecture in rice was selected as a target gene for base editing. The sequencing results showed expected T/C substitutions at the target region achieving an efficiency of 26%. Nine predicted off-target sites did not have any base editing events. These results indicate the specificity and efficiency of adenine base editors in rice. The above studies demonstrate the application of base editors toward rice improvement (**Figure 2**).

### Generation of Rice Mutant Libraries

With the availability of whole genome sequence in rice, the greater challenge is to analyze and assign functions to all the predicted genes in the genome which could be priority in rice functional genomics studies (Wang et al., 2013). With the advent of CRISPR/Cas9 system, development of such mutant libraries has become more conducive. Recently, a high quality large-scale CRISPR/Cas9 mutant library has been constructed in rice for studying gene functions (Meng et al., 2017). The researchers selected 12,802 genes highly expressed in rice shoot base tissue and 25,604 corresponding sgRNAs to generate a large-scale mutant library. More of such rice mutant libraries are expected to be constructed in near future that could play significant role in identifying gene function and accelerating genetic improvement.

## Genome Editing in Rice Improvement

The first group of studies that demonstrated the applications of genome editing in plants was reported in the model species *A. thaliana* and *N. benthamiana* (Feng et al., 2013; Li et al., 2013; Nekrasov et al., 2013), as well as crops such as rice (Jiang et al., 2013; Miao et al., 2013; Shan et al., 2013; Wang et al., 2016), wheat (Shan et al., 2013; Wang et al., 2014), maize (Char et al., 2016), potato (Butler et al., 2015), and tomato (Pan et al., 2016). In the last several years, CRISPR/Cas9 has been used to produce new rice varieties with improved traits, including enhance disease resistance and other stress tolerance, nutritional improvement, and increased yield (**Table 1**).

**Table 1:** List of genes targeted by genome editing tools for rice improvement.

Application perspectives	Targeted gene	Genome editing strategy	Molecular functions	Reference
Yield and quality improvement	*LOX3*	TALENs	Enhanced storage tolerance	Ma L. et al., 2015
	*GW2*, *GW5*, and *TGW6*	CRISPR/Cas 9	Improvement of grain weight	Xu et al., 2016
	*Hd2*, *Hd4*, and *Hd5*	CRISPR/Cas 9	Early maturity of rice varieties	Li X. et al., 2017
	*CSA*	CRISPR/Cas 9	Photoperiod controlled male sterile lines	Li M. et al., 2016
	*Gn1a*, *DEP1*, *GS3*, and *IPA1*	CRISPR/Cas 9	Improvement of grain number, panicle architecture, grain size, and plant architecture	Li M. et al., 2016
	*CCD7*	CRISPR/Cas 9	Increased tiller number	
	*PYLs*	CRISPR/Cas 9	Improved growth and productivity	Miao et al., 2018
	*OsBADH2*	TALENs	Enhanced fragrance	Shan et al., 2015
	*BADH2*	CRISPR/Cas 9	Enhanced fragrance	Shao et al., 2017
Biotic stress tolerance	*OsSWEET13*	TALENs	Enhanced resistance to bacterial blight	Li et al., 2012
	*OsSWEET13*	TALENs	Enhanced resistance to bacterial blight	Zhou et al., 2015
	*OsSWEET13*	TALENs	Enhanced resistance to bacterial blight	Blanvillain-Baufum et al., 2017
	*Os09g29100*	TALENs	Enhanced resistance to bacterial leaf streak	Cai et al., 2017
	*OsERF922*	CRISPR/Cas 9	Enhanced resistance to blast disease	Wang et al., 2016
Abiotic stress tolerance	*BEL*	CRISPR/Cas 9	Herbicide resistant	Xu et al., 2014
	*OsEPSPS*	CRISPR/Cas 9	Glyphosate resistant	Li J. et al., 2016
	*OsALS*	TALENs	Herbicide resistant	Li T. et al., 2016
	*ALS*	CRISPR/Cas 9	Herbicide resistant	Sun et al., 2016
	*C287*	Base editing	Herbicide resistant	Shimatani et al., 2017
	*Os SAPK2*	CRISPR/Cas 9	Drought tolerance	Lou et al., 2017
Nutritional improvement	*OsNRAMP5*	CRISPR/Cas 9	Low cadmium content	Tang et al., 2017
	*SBEIIb* and *SBEI*	CRISPR/Cas 9	Generation of high amylose rice	Sun et al., 2017
	*OsPDS*, *OsSBEIIb*	Base editing	Nutritional improvement	Li J. et al., 2017
Stomatal density	*OsEPFL9*	CRISPR/Cas 9 and CRISPR/Cpf1	Regulates leaf stomatal density	Yin et al., 2017
Nitrogen use efficiency	*NRT1.1B* gene	Base editing	Enhance nitrogen use efficiency	Lu and Zhu, 2017
Senescence and death	*OsCDC48*	Base editing	Regulate senescence and death	Zhong et al., 2017


### Yield and Quality Improvement

Yield and quality are typical quantitative traits governed by multiple genomic loci. In rice, yield is mainly determined by three major components – number of panicles per plant, number of grains per panicle, and grain weight (Xing and Zhang, 2010). Rice yield has been improved by knocking out genes including *GS3*, *DEP1*, *GS5*, *GW2*, *Gn1a*, and *TGW6* that are known to be negative regulators of grain size and number and grain weight (Zhang et al., 2017). Four genes *Gn1a*, *DEP1*, *GS3*, and *IPA1* have been independently edited using the CRISPR/Cas9 system resulting in expected phenotypes such as enhanced grain number, dense erect panicles, and larger grain size (Li M. et al., 2016). In another study, the simultaneous mutation of three grain weight related genes – grain weight 2 (GW2), GW5, and *thousand grain weight* 6 (TGW6) using CRISPR/Cas9 resulted in 29.3% increase in 1000 grain weight in the triple null mutant (Xu et al., 2016). This suggests that pyramiding null mutants of major yield related genes in a single cultivar *via* multiplex gene editing would be crucial in regulating the yield components of rice. Similarly, rice heading date is also an important agronomic trait that contributes to rice yield. CRISPR/Cas 9-mediated multiplex genome editing was used for developing a high efficiency breeding approach by targeted mutagenesis of three major genes (*Hd 2*, *Hd 4*, and *Hd 5*) known to negatively affect heading date in rice (Li X. et al., 2017). Results revealed that the heading date of mutated derivative from nine rice lines was significantly shortened to different extent.

Hybrid rice with a yield advantage of 10–20% over inbred lines significantly contributes to rice production. CRISPR/Cas9 system have been utilized to induce specific mutations in a thermo-sensitive gene *TMS5* to develop 11 new TGMS *indica* rice lines within only 1 year (Zhou et al., 2016). In a similar experiment, CRISPR/Cas9 technology was used for targeting *Carbon Starved Anther (CSA)* gene, a prominent locus that display male sterility under short day conditions and male fertility under long day conditions in japonica rice to develop two reverse PGMS lines *9522csa* and *JY5Bcsa* and one rP(T)GMS145 line *KY131csa-4* (Li Q. et al., 2016). Thus, CRISPR/Cas technology can accelerate TGMS line breeding which is the first step toward large scale application in two-line hybrid rice breeding.

Grain deterioration during storage compromises the quality and seed longevity in rice causing serious economic losses. Rice seed storability is mainly affected by lipoxygenases (LOXs) enzymes which catalyze the dioxygenation of polyunsaturated fatty acids to form hydroperoxide. Three of the 14 LOX proteins (LOX1, LOX2, and LOX3) have distinct functions in rice acting as negative regulators of seed longevity. TALEN technology has been used to develop a modified ligation-independent cloning method (LIC) for efficient mutation of the LOX3 gene using a pair of TALEN monomer (Ma L. et al., 2015). The seeds of LOX3 knockout lines exhibited improved storability. Additionally, fragrant rice is favored worldwide and has a higher market value due to its agreeable smelling quality. The suppression of abetaine aldehyde dehydrogenase (BADH2) protein induces the synthesis of 2-acetyl-1-pyrroline content (2AP) which is the major fragrance compound in rice. TALEN induced mutation in the *OsBADH2* gene has resulted in 35–75% increase in 2AP content among the gene edited lines (Ma L. et al., 2015). Recently, CRISPR/CAS9 has been used to edit the fragrant gene *Badh2* in the indica rice line, Zhonghua 11 (Shao et al., 2017). The mutated line contained an additional T base in the first exon of *Badh2* and resulted in increased amount of 2AP and enhanced fragrance in rice.

Growth and yield are also controlled by several phytohormones and their overlapping signaling networks. Abscisic acid (ABA), a crucial phytohormone, is perceived by the soluble pyrabactin resistance 1 (PYR1)/PYR1-like (PYL)/regulatory components of the ABA receptor (RCAR) family proteins. CRISPR/Cas9 technology was efficiently used to edit group I (PYL1–PYL6 and *PYL12*) and group II (*PYL7–PYL11* and *PYL13*) *PYL* genes in rice which led to increased growth and productivity in rice (Miao et al., 2018). Mutation of group I genes (*PYL1–PYL6* and *PYL12*) promoted rice growth and among them pyl1/4/6 exhibited the most robust growth and improved grain productivity, while maintaining near-normal seed dormancy and other agronomic traits. These results provide another genetic strategy to improve rice productivity.

### Nutritional Improvement

Rice is the major source of nutrients and contributes up to 70% of daily calories for more than half of the world population. High amylase content (AC) and resistant starch (RS) improves human health and reduce the risk of serious diseases including hypertension, diabetes, and colon cancers (Chen et al., 2012). Therefore, there is an increasing need to develop rice varieties with high AC and RS to meet the growing challenges in nutrition for public health. The CRISPR/Cas9 technology has been successfully used to create high amylose rice by targeting two rice branching enzyme (SBE) *SBEI and SBEIIb* (Sun et al., 2017). While the *sbeI* mutants and wild types did not reveal any variations, *sbeII* mutants showed significant increase in AC and RS content to as higher as 25 and 9.8, respectively. This suggest that *SBEII* plays a significant role in determining the fine structure and nutritional properties of starch and CRISPR/Cas9-mediated editing of *SBEIIb* would be crucial in the development of high amylase and RS rice varieties.

Genome editing can also be used to modify rice genes resulting in non-toxic and healthier varieties. Cadmium (Cd) is a highly toxic heavy metal which causes serious health effects in people who consume rice as a staple food. The Cd content in *indica* cultivars is comparatively more as compared to *japonica* rice cultivars and need to be controlled for food safety (Arao and Ishikawa, 2006; Grant et al., 2008). Several measures such as soil treatment, phytoremediation, field flooding, and charcoal application have been undertaken to reduce the Cd level but they were only effective to some extent. Conventional approaches for developing low Cd rice cultivars is highly challenging and new strategies for Cd free rice lines are imperative for public health. CRISPR/Cas9 editing system has been recently used to develop *indica* rice lines with low Cd accumulation by knocking out the metal transporter gene *OsNramp5* (Tang et al., 2017). Field trials of the mutated *indica* rice lines showed that the Cd concentration in *OsNramp5* grains was consistently less than 0.05 mg/kg, as compared to the high Cd concentrations from 0.33 to 2.90 mg/kg in grains of wild-type *indica* rice without affecting the plant yield. Genome editing systems are expected to be used in near future to minimize multiple heavy metal contamination risks in rice grains.

### Biotic Stress Tolerance

Diseases caused by biotic agents including bacteria, fungi, viruses, and insects are the main reason for rice yield loss and poor product quality (Heinrichs and Muniappan, 2017). Several disease related genes have been mutated in the recent times using the genome editing approaches to increase disease resistance in rice. Bacterial leaf blight (BLB), a widespread vascular rice disease caused by *Xanthomonas oryzae pv. oryzae* (*Xoo)*, is a major threat for global food security. Conventional and molecular breeding approach has always been an effective approach for disease management and considerable efforts have been made toward disease resistance against *Xoo* (Pradhan et al., 2015; Das et al., 2018). However, the rapid emergence of new virulent pathotypes has led to the rise of advanced approaches for combating blight disease. Transcription activator-like effectors (TALEs), the type III effector proteins from *Xanthomonas* species, usually target the SWEET gene family, the sugar transporters that release the sugar into the apoplast of rice cells (Cohn et al., 2014). TAL effectors AvrXa7 or PthXo3 of *Xoo* target and activate the sucrose-efflux transporter gene *OsSWEET14* and thus transport the sugars from the plant cell to satisfy the pathogen needs (Antony et al., 2010). TALEN technology was used to disrupt the bacterial protein binding sequence in the promoter of *OsSWEET14* for conferring resistance against bacterial blight (Li et al., 2012). Similarly, CRISPR/Cas9 technology was used to construct a null mutation in *OsSWEET13* to prevent its neutralization by the TAL effector gene *pthXo2* leading to improved resistance toward bacterial blight disease in *indica* rice, IR24 (Zhou et al., 2015). Besides the PthXo3, additional TALEs such as AvrXa7, TalC, and Tal5 also target *OsSWEET14*, making it a major susceptibility factor during *Xoo*–rice interactions (Blanvillain-Baufum et al., 2017). Most recently, TALEN technology was used to modify the *EBEtal7* binding site in the *Os09g29100* gene promoter to reduce Tal7 binding, which could potentially reduce BLB disease severity in rice (Cai et al., 2017). All these approaches are quite promising in engineering rice cultivars with reduced susceptibility to *Xoo.*

Rice blast caused by ascomycetes fungus *Magnaporthe oryzae* is the most destructive disease of rice in all rice growing countries and has threatened the global food security (Zhang et al., 2014a). Blast almost results in 60–100% yield losses in large rice producing areas under favorable environmental conditions (Kihoro et al., 2013). Over the past few decades, tremendous efforts have been made to develop blast resistant cultivars by the use of advanced molecular and genomic tools. Conventional host resistance breeding has played an important role in developing novel blast resistance (Fukuoka et al., 2014; Ashkani et al., 2015). However, it is highly time consuming and laborious. Furthermore, the emergence of new pathogenic variability often leads to breakdown of resistance cultivars. Thus, recent development of engineered nucleases could be a suitable alternative for enhancing the resistance of rice to *M. oryzae*. CRISPR/Cas9-targeted knockout of ERF transcription factor gene *OsERF922* has demonstrated enhanced resistance to rice blast (Wang et al., 2016). Targeted mutagenesis revealed insertion or deletion at the target site and the mutation frequency was up to 42% in T_0_ plants. Phenotypic assessment of six T2 homozygous mutant lines revealed that the number of blast lesions was significantly decreased as compared to wild-type plants. This suggests that CRISPR/Cas9 is a useful approach for enhancing blast resistance in rice.

Rice tungro disease (RTD) is another important rice disease that severely affects rice production mostly in Asian countries. It is caused by the interaction of two different viruses, namely, rice tungro spherical virus (RTSV) and rice tungro bacilliform virus (RTBV). Extensive breeding for resistance development and evaluation of resistant near isogenic lines have shown that the translation initiation factor 4 gamma (*eIF4G*) gene is responsible for RTSV resistance and the YVV residues of eIF4G are known to be associated with the reactions to RTSV (Lee et al., 2010). A CRISPR/Cas9-mediated editing of *eIF4G* gene has been reported in the RTSV susceptible rice variety, IR64 as an attempt to develop new source of resistance to RTD (Macovei et al., 2018). Novel *eIF4G* alleles were transmitted to T1 and T2 generations with no detectable mutations in the closest off-target sites. These RTSV-resistant plants with the novel *eIF4G* alleles can be used as valuable materials to develop more diverse RTSV-resistant varieties.

### Abiotic Stress Tolerance

The rice bentazon sensitive lethal (BEL) gene confers resistance to bentazon and sulfonylurea herbicides and the loss-of-function mutant *bel* is sensitive to the herbicides (Pan et al., 2006). The *bel* mutant is often used in the selection of seed contamination in a two-line hybrid rice production system. Even though *BEL* is significant in determining hybrid rice production safety, its application is always restricted due to limited natural genetic resources. Therefore, a CRISPR/Cas9-based mutation of *BEL* gene was assayed in rice using the *Agrobacterium*-mediated gene transfer (Xu et al., 2014). Stable CRISPR/Cas9 transformants demonstrated 2–16% mutagenic efficiency while the phenotypic analysis revealed that the biallelic mutated transgenic plant was sensitive to bentazon.

Recently, genome editing-based mutations has been introduced within the ALS gene to produce herbicide tolerant rice varieties (Li T. et al., 2016; Sun et al., 2016). As a proof of concept, Li T. et al. (2016) used a gene replacement strategy using the TALEN-based HR in rice and produced double point mutations in rice *OsALS.* The mutation efficiency was 6.3% with the entire stable mutant displaying strong herbicide tolerance. In a similar experiment, CRISPR/Cas9-mediated HR was performed to introduce multiple discrete point mutations in the rice *ALS* gene (Sun et al., 2016). Phenotypic screening showed that the wild-type plant dyed after 36 days of bispyribac sodium (BS) spraying while the edited lines exhibited tolerance to BS and grew normally. Thus, genome editing system could be efficiently used for the generation of homozygous herbicide tolerance rice plants within one generation.

Rice seedlings are sensitive to low temperature, especially at the seedling stage. Thus, improvement of cold tolerance can significantly enhance rice productivity. TIFY1b, a transcription factor, is one of the cold tolerant involving gene discovered in rice. CRISPR/Cas9 technology was employed to edit the *TIFY1b* and its homology gene *TIFY1a* (Huang et al., 2017). Site-specific mutations were observed in T_0_ rice plants. The study indicates the *TIFY1* mutant lines could be further used to investigate the role of *TIFY1* genes in rice adaptation to low temperature.

## Conclusion and Future Implications

In conclusion, gene editing technologies, particularly the CRISPR/Cas9 system, hold a greater significance in defining plant research in the recent times. It has truly emerged as the most effective tool for crop improvement owing to its ability to create mutations at desired targets in the genome with greater accuracy, efficiency, and simplicity. A major advantage of this process lies in the fact that the transgenes causing genetic modification can be easily eliminated from the genome through genetic segregation resulting in no differences between the gene-edited plants and those developed through conventional breeding. The development of CRISPR–Cpf1 system and base editing by far holds greater promise for editing rice genome with much more precision and efficiency. Furthermore, genome editing-based epigenetic regulation through the manipulation of DNA methylation and histone modification also holds greater promise in crop improvement as such modifications can be inherited into plant off springs without any change in the genomic sequence. A recent study involving CRISPR/dCas9 fused with DNA methyl transferase 3a (DNMT3a) induced DNA methylation in the target regions of the mammalian cells (Liu et al., 2016). Although such epigenome editing tools are yet to be available in plant system, their development in plant system will add in new dimension to the genome editing-based improvement of rice and other crops.

Nevertheless, there are still some challenges in application of genome editing. Mitigating these challenges in rice and other crops can promote efficient application of this admirable technology in crop improvement. The first challenge is to mitigate the PAM requirement for CRISPR-based genome editing systems. A unique PAM site required by SpCas9 is a major factor that determines CRISPR/Cas specificity (Hsu et al., 2014). The requirement of a specific PAM is quite stringent and might affect the efficiency of genome editing by limiting the sequences to be addressed. However, the alternative PAM sequences (3′ NAG and NGA) (Zhang et al., 2014b; Kleinstiver et al., 2016) and orthogonal Cas9 variants StCas9 and SaCas9 (Kaya et al., 2016) have widen the applications of genome editing in plants. Most recently, a study has revealed that the wild-type SpCas9 is robust in recognizing both 5′-NAG-3′ and 5′-NGG-3′ PAMs in rice (Meng et al., 2018). The study further reported that the usage of 5′-NAG-3′ alone or together with 5′-NGG-3′ results in efficient genome editing in rice with relatively low off-target effects. For further expanding the range of genome editing in plants, the VQR and VRER variants of CRISPR/Cas9 system have been developed (Hu et al., 2016). In a recent study, researchers have made attempts to increase the editing efficiency of VQR variants in rice (Hu et al., 2018). The newly modified CRISPR–Cas9–VQR system is particularly suitable for efficient genome editing with 5′-NGA-3′ PAMs. Most recently, a new SpCas9 variant (xCas9) has been reported that can recognize a broad range of PAM sequences including NG, GAA, and GAT (Hu et al., 2018). All these findings will help in expanding the scope of genome editing by using several engineered Cas9 variants with different PAM specificities. However, not all of these Cas9 variants are effective in plants and there is a lot of scope for development of more Cas9 variants which can recognize a wide range of PAM sites and expand the range of genome editing in cereal crops, especially in rice.

The second challenge is to significantly increase efficiency in gene replacement editing. Because more desired traits for crop breeding relay on gain-of-function mutation, precision editing by sequence replacement and fragment knock-in *via* HR have more important implications for crop improvement. However, the gene replacement editing is still difficult because the efficiency of HR is very low in plants. There is a need for higher efficient HR-based genome editing besides maintaining a balance between the NHEJ and HR pathways. Optimization of effective delivery methods of the donor template DNA may mitigate the challenge in gene replacement editing.

The third challenge is to make the edited rice and other crops success early in farmer’s field. Most of the works on genome editing reported so far are only proofs of concept in confined environments. Uncertainties about the edited plant’s performance in natural environmental conditions might exist. More field trails of the edited plants will make this technical issue clear. Another factor limiting the adoption of edited rice in farmer’s field is the biosafety regulation rules. Although the USDA has exempted the application of strict GMO regulations in many CRISPR-edited crops including waxy corn, flavored tomato, and mushroom (Waltz, 2016), the Court of Justice of the European Union has recently judged that organisms created using genome editing techniques are to be regulated as GMOs (Callaway, 2018). This might affect the decision of rice growing countries on the regulation of edited rice. Anyhow, genome editing technology can technically create modified crop plants with no differences from those developed through conventional breeding. CRISPR/Cas9 and associated genome editing tools have brought in a revolutionary change in rice improvement which is crucial for meeting the demands and ensuring the requirement of rice for future generations.

## Author Contributions

The manuscript was written by RM and RJ under the supervision of KZ. The manuscript was critically revised by KZ. All the authors have read and approved the final manuscript.

## Conflict of Interest Statement

The authors declare that the research was conducted in the absence of any commercial or financial relationships that could be construed as a potential conflict of interest.
